# PATTERNS OF MULTIPLE CAREGIVING AND DEPRESSIVE SYMPTOMS AMONG MIDDLE-AGED AND OLDER ADULTS IN CHINA

**DOI:** 10.1093/geroni/igae098.1833

**Published:** 2024-12-31

**Authors:** Xinyi Zhao, Vivian Weiqun Lou

**Affiliations:** Peking University, Beijing, Beijing, China (People’s Republic); The University of Hong Kong, Hong Kong, China (People’s Republic)

## Abstract

**Objectives:**

With extended life expectancy, middle-aged and older adults have become more involved in multiple caregiving. This study aimed to investigate the patterns of multiple caregiving, and whether these patterns predicted follow-up depressive symptoms.

**Methods:**

Data were obtained from the 2011–2015 waves of the China Health and Retirement Longitudinal Study. A total of 11,422 participants aged 50 and older in 2011 were included in the latent class analysis to identify the patterns of multiple caregiving. A regression model was used to analyze the association between baseline caregiving patterns and subsequent depressive symptoms.

**Results:**

Four caregiving patterns were established: “part-time care for all”, “parent care and full-time grandchild care”, “part-time spouse care and full-time grandchild care” and “full-time spouse care”. Compared with the “part-time care for all” group, the “full-time spouse care” group and “part-time spouse care and full-time grandchild care” group reported significantly greater depressive symptoms four years later.

**Conclusion:**

The use of categorizing multiple caregiving could vividly describe middle aged and older adults’ engagement in caregiving activities and provide a comprehensive prediction of mental health outcomes. Social services should be tailored to sandwiched caregivers in certain caregiving patterns.

